# Recent Advances in the Diagnosis and Treatment of Influenza Pneumonia

**DOI:** 10.1007/s11908-012-0257-5

**Published:** 2012-04-03

**Authors:** Lucia Marzoratti, Hernán A. Iannella, Victoria Fernández Gómez, Sandra B. Figueroa

**Affiliations:** 1 CEMIT (Centro Médico Investigadores Tucumán), San Miguel de Tucumán, Tucumán Argentina; 2 Pulmonary Medicine Division, Hospital de Clínicas, Universidad de Buenos Aires, Buenos Aires, Argentina

**Keywords:** Influenza pneumonia, Diagnosis, Treatment, Prevention, Swine influenza, Avian influenza, H1N1, Pandemic

## Abstract

A potentially fatal complication of influenza infection is the development of pneumonia, caused either directly by the influenza virus, or by secondary bacterial infection. Pneumonia related to the 2009 influenza A pandemic was found to be underestimated by commonly used pneumonia severity scores in many cases, and to be rapidly progressive, leading to respiratory failure. Confirmation of etiology by laboratory testing is warranted in such cases. Rapid antigen and immunofluorescence testing are useful screening tests, but have limited sensitivity. Confirmation of pandemic H1N1 influenza A infection can only be made by real-time reverse-transcriptase polymerase chain reaction (rRT-PCR) or viral culture. The most effective preventive measure is annual influenza vaccination in selected individuals. Decisions to administer antiviral medications for influenza treatment or chemoprophylaxis should be based upon clinical and epidemiological factors, and should not be delayed by confirmatory laboratory testing results. Neuraminidase inhibitors (NI) are the agents of choice.

## Introduction

Influenza is acute respiratory illness caused by influenza A or B viruses with seasonal circulation during the winter months. However, outbreaks of novel recombinant strains that take place in animals have produced, along the years, many worldwide outbreaks with serious public health issues. Although normally a self-limited process in the general population, high risk groups for complications and death have been identified (Table 1). During an outbreak, in otherwise healthy subjects the diagnosis of influenza infection can be made confidently based on clinical manifestations alone. However, in certain situations, such as sporadic cases, in patients at an increased risk for complications, or in hospitalized patients with severe pulmonary compromise, confirmation of etiology by laboratory testing is required to guide treatment and for surveillance purposes [1••, 2••]. A potentially fatal complication of influenza infection is the involvement of the lower respiratory tract caused directly by the influenza virus, and the development of secondary bacterial pneumonia. Novel recombinant influenza A strains carry the risk for more severe disease and have the potential to cause widespread illness and a large number of deaths, regardless of age or previous health status. Examples of this are the H5N1 “avian” influenza A outbreak since 2004, and the more recent H1N1 “swine” influenza A pandemic in 2009, both of which have prompted the development of quick and reliable laboratory test in an effort to optimize their management and reduce morbidity and mortality. In this article, we review the latest available data for the diagnosis of influenza lower respiratory tract infection, as well as for treatment and prevention strategies.

**Table 1:** Persons at higher risk for influenza complications

Children <2 years
Adults ≥65 years
Women who are pregnant or postpartum (within 2 weeks after delivery)
Individuals with chronic medical conditions
Pulmonary disease (including asthma)
Cardiovascular disease (except isolated hypertension)
Active malignancy
Chronic renal insufficiency
Chronic liver disease
Diabetes mellitus
Hemoglobinopathies (including sickle cell disease)
Immunosuppression ^1^
Any neurologic condition that can compromise handling of respiratory secretions ^2^
Native Americans and Alaska Natives
Morbidly obese persons (body-mass index ≥40)
Residents of nursing homes and other chronic-care facilities

(1) Including HIV infection (particularly if CD4 <200 cells/microL), organ or hematopoietic cell transplantation and inflammatory disorders treated with immunosuppressants

(2) Including disorders of the brain, spinal cord, peripheral nerve, and muscle such as cerebral palsy, seizure disorders, stroke, intellectual disability, moderate to severe developmental delay, muscular dystrophy, or spinal cord injury

*Adapted from the recommendations of the Advisory Committee on Immunization Practices (ACIP)* [40••]

## Clinical and Radiological Diagnosis

Signs and symptoms of upper and/or lower respiratory tract infection, along with systemic involvement in the form of fever, myalgia, and headache, are usually the main presenting features of the disease. In the context of an outbreak, otherwise healthy subjects presenting with a self-limited acute febrile respiratory illness usually require no further diagnostic procedures. In two retrospective studies that examined which clinical signs and symptoms are most predictive of influenza infection in patients with influenza-like illness, cough and fever were the only symptoms significantly associated with a positive PCR test for influenza [3, 4]. In another study, no isolated symptom or sign was able to accurately predict influenza infection, though the absence of fever, cough and nasal congestion significantly decreased its likelihood [5]. In general, patients diagnosed with pandemic H1N1 influenza A virus had similar signs and symptoms compared to those with seasonal influenza. However, these patients had gastrointestinal manifestations more frequently [6, 7], were more likely to have pneumonia [8], and also had higher rates of extrapulmonary complications, intensive care unit admission, and death [9].

Pneumonia is the most frequent and severe complication of influenza, most commonly presenting in high risk patients (Table 1). Primary influenza pneumonia represents direct lung involvement by influenza virus, and should be suspected in non-resolving influenza infections. Typically, primary influenza pneumonia presents in chest x-rays with bilateral reticular or reticulonodular opacities. Less frequently, focal areas of consolidation can be seen, particularly in the lower lobes. High-resolution computed tomography may show ground glass opacities with or without multifocal peribronchovascular and subpleural consolidation [10].

The cytopathic effect of the influenza virus on the tracheobronchial epithelium may predispose to secondary bacterial pneumonia [11, 12]. Secondary bacterial pneumonia must be suspected whenever there is an exacerbation of fever and respiratory symptoms after initial improvement in a patient diagnosed with acute influenza. Leukocytosis, instead of a normal or low white blood cell count, and lobar consolidation on chest imaging, instead of the diffuse pattern that is typical of viral pneumonia, are also suggestive [13].

In an observational study of 543 hospitalized patients with H1N1 influenza A infection in Spain, 43 % of the 243 patients in which chest radiographs were performed had pneumonia, 83 % of the 210 patients who had microbiologic confirmation had primary influenza pneumonia, and the remaining 17 % had concomitant secondary bacterial pneumonia. Bilateral pneumonia occurred in 48.3 % of patients; *Streptococcus pneumoniae* being the most frequent pathogen [14]. Several reports have identified methicillin-resistant *Staphylococcus aureus* (MRSA) as the etiologic agent for severe community acquired pneumonia (CAP) in otherwise healthy young patients with influenza [15–17]. In another study that investigated the incidence of community-acquired MRSA pneumonia in H1N1 influenza patients, 50 patients of 4491 (1 %) laboratory-confirmed pandemic influenza A (H1N1) cases had a bacterial respiratory tract pathogen. The most commonly cultured organisms were *S. pneumoniae* (16 patients), *S. aureus* (13 patients) and *Haemophilus influenzae* (9 patients); MRSA was detected in only 2 patients [18]. In contrast, among 838 children and adolescents admitted to 35 intensive care units in the U.S. with confirmed or probable severe H1N1 influenza A infection, 48 % of the 71 patients with suspected diagnosis of early *S. aureus* coinfection had MRSA [19].

Non-seasonal influenza infections have specific clinical manifestations. Pneumonia related to the 2009 H1N1 influenza A pandemic was also found in many cases to be rapidly progressive, leading to respiratory failure and ARDS [20•, 21•]. Additionally, the risk for complications and death due to that pandemic influenza was found to be underestimated by commonly used pneumonia severity scores [22•, 23]. Avian influenza (H5N1) frequently presents as severe primary pneumonia that often progresses rapidly to the acute respiratory distress syndrome (ARDS), having caused high rates of death, especially among infants and young children in Southeast Asian countries [24].

## Laboratory Testing

In certain situations, confirmation of etiology by laboratory testing is required in order to guide the initiation and duration of antiviral therapy, and for the implementation of infection control measures and surveillance. Other benefits of influenza virus detection are the reduction of inappropriate antibiotic use, decreased length of stay in emergency departments, and fewer additional laboratory studies, all leading to a reduction in health care costs [1••]. The Centers for Disease Control and Prevention (CDC) and the Infectious Diseases Society of America (IDSA) have published guidelines to better define patients who should undergo influenza testing [1••, 2••]. The available methods include immunological techniques (i.e. rapid antigen-based tests, immunofluorescence assays, serologic testing), molecular techniques (i.e. reverse-transcriptase polymerase chain reaction [RT-PCR]), and microbiological techniques (i.e. viral cultures). While RT-PCR has the highest sensitivity and specificity, rapid antigen and immunofluorescence testing, though very useful as initial screening tests, are considerably less sensitive. Besides, the sensitivity and specificity of any of these techniques will vary depending on the laboratory equipment, personnel expertise, the timing of recollection, and the appropriate handling of the samples [1••, 2••]. Respiratory specimens can be obtained by many different methods including throat swabs, nasal aspirates, and nasopharyngeal swabs, aspirates and washing [2••]. In mechanically ventilated subjects, more invasive maneuvers such as endotracheal aspirates and bronchoscopic or non-bronchoscopic bronchoalveolar lavage (BAL) may be required in order to obtain adequate lower respiratory tract samples. While rapid antigen tests, immunofluorescence and RT-PCR all can yield results quick enough to guide point-of-care clinical decision-making, serologic tests and viral cultures provide retrospective diagnosis, availability only in reference laboratories, and usefulness when confirming screening test. For this reason, they are normally reserved for epidemiological and research purposes (Table 2).

**Table 2:** Influenza testing methods

Method	Test Time	Specimen	Sensitivity	Specificity	Distinguishes influenza A from B	Influenza A subtypes
RITD	15 min.	Respiratory samples ^1^	+	+++	yes ^2^	no
Immuno-fluorescence ^3^	1-4 h.	Respiratory samples ^1^	++	+++	yes	no
RT-PCR ^4^	1-6 h.	Respiratory samples ^1^	+++	++++	yes	yes
Viral culture ^5^	1-10 d.	Respiratory samples ^1^	++	+++++		
Serologic tests	2 w.	Serum ^6^	n/a	n/a	yes	yes

*RIDT* rapid influenza diagnostic tests, *RT-PCR* reverse-transcriptase polymerase chain reaction

(1) Appropriate respiratory samples vary for each method and should be obtained according to the manufacturer’s specifications

(2) Some commercially-available RIDT distinguish between influenza A and B while others do not

(3) Direct (DFA) or indirect (IFA) antibody staining

(4) Conventional gel-based PCR, real-time RT-PCR, and multiplex PCR

(5) Rapid viral cultures (shell vials) can yield results in 1-3 days, compared to conventional isolation in cell culture (3-10 days)

*Adapted from the IDSA and CDC guidelines* [1••**,** 2••**]**

## Rapid Influenza Diagnostic Tests

Rapid influenza diagnostic test (RIDT) are designed to detect influenza A and B nucleoproteic antigens in respiratory specimens, with results expressed qualitatively as positive or negative in no more than 15 min. Many different FDA-approved tests are available; while some assays are capable of distinguishing between influenza A or B viruses, none can distinguish between pandemic and seasonal strains of influenza A [1••, 2••]. Compared to reference methods (i.e. RT-PCR and viral culture), they have low sensibility (10 % to 80 %) and high specificity (95 % to 100 %), and there is also great variability between the different commercially available kits [25–27]. Sensitivity appears to be somewhat lower for influenza B than that seen for influenza A [26]. Also, the reported sensitivity for rapid tests is higher for nasopharyngeal samples than for throat swabs [27]. There are two main factors that influence RIDT’s negative predictive value. First, negative results obtained during periods of high viral activity in the community are more likely to be false negatives; alternatively, false positives, though much less frequent, are more likely during periods of less viral circulation. Second, respiratory samples collected within the first 48 to 72 h of symptom onset positively influence RIDT’s sensitivity. For these reasons, when rapid tests are negative, confirmation by means of RT-PCR or viral culture should be considered [1••, 2••]. Compared to RT-PCR, the sensitivity of RIDTs for detecting novel influenza A (H1N1) was equal to or lower than the sensitivity to detect seasonal influenza viruses [29–32], so RIDT results need to be interpreted with caution when evaluating patients suspected of having pandemic H1N1 influenza A.

## Immunofluorescence

Direct (DFA) or indirect (IFA) immunofluorescence antibody staining techniques are capable of detecting influenza A and B viruses, and distinguish the viruses from each other as well as from other respiratory viruses [1••, 2••]. They have levels of sensitivity and specificity that come close to those of RT-PCR [28, 33], and results are often available in a few hours [1••, 2••]. Although these tests have improved sensitivity over RIDT, they are more technically complex and require expertise in obtaining quality respiratory samples (Table 2).

## Reverse-Transcriptase Polymerase Chain Reaction

This is the reference influenza detection method and has the highest sensitivity and specificity [34, 35]. Several modalities of RT-PCR have been designed: conventional gel-based PCR (cRT-PCR), multiplex PCR (mRT-PCR), and real-time RT-PCR (rRT-PCR). They can differentiate between influenza types (A or B) and subtypes (including pandemic H1N1 influenza and avian H5N1 influenza), and results are available in 2–6 h (although due to transportation of batched specimens to reference centers for processing, it may take longer for results to be available). During the 2009 H1N1 influenza A pandemic, it became clear that rapid case identification was essential for timely management of patients and for adequate public health actions to be taken. To answer to this threat, the CDC optimized the previously developed rRT-PCR procedures for detection of the A/H1N1 2009 pandemic influenza virus [36•]. Yang et al. compared the performance of 12 rRT-PCR primer-probe sets designed for detecting the hemagglutinin (HA) or the neuraminidase (NA) gene of the pandemic influenza A/H1N1 2009 virus, using the primer-probe set developed at the CDC as reference. They found that although all primer-probe sets had specificity levels as high as 98.4 % to 100 %, some of the primer-probe sets had better specificity, sensitivity, and amplification efficiency than others, and that a combination of primer-probe sets targeted to the HA and NA genes had higher detection sensitivity than those targeting HA or NA individually [37]. In another study, Lam et al. showed that although rRT-PCR assays can be 10-fold more sensitive than cRT-PCR, newly developed cRT-PCR assays targeting the HA gene are a reliable alternative for laboratories where a rRT-PCR machine is not available [38]. Sensitivity and specificity of these assays also depend on the type of respiratory sample employed. For example, in a Spanish study that assessed the utility of rRT-PCR for the diagnosis of the novel influenza A/H1N1 virus, the authors reported that the diagnostic yield of combined nose and throat swabs was higher than that of nasopharyngeal aspirates [39].

## Viral Cultures and Serologic Tests

Even the fastest viral cultures techniques can take days to demonstrate influenza cytopathic effects. Since they are not suitable for initial clinical management, their utility during an influenza outbreak is to confirm some negative test results from RIDT and immunofluorescence. Viral cultures also provide information about circulating influenza strains and its subtypes; this information is required for next season vaccine production, for surveillance of the emergence of new influenza A strains, and for the detection of antiviral resistance. Similarly, serologic tests (i.e., hemagglutinin inhibition, ELISA, complement-fixation) that demonstrate a four-fold increase in serum antibody titers between acute and convalescence phases of the disease, are only useful for retrospective diagnosis or research purposes (Table 2).

## Treatment and Prevention

Annual immunization is the most important preventive measure [40••]. However, two classes of antiviral drugs are available and play an important role in the treatment and prevention of influenza [41••]: the neuraminidase inhibitors (NI), oseltamivir and zanamivir, which are active against both influenza A and B viruses; and the M2 inhibitors, amantadine and rimantadine, which are active against all influenza A strains, but have no activity against influenza B viruses. In general, the duration for therapy with an NI is 5 days, and with the M2 inhibitors is three to 5 days.

## Pharmacology

NI are sialic acid analogs that competitively inhibit neuraminidase on the surface of both influenza A and B, thus interfering with the release of virus from infected cells. Oseltamivir phosphate is an orally bioavailable prodrug that is rapidly absorbed from the gastrointestinal tract and is converted by hepatic esterases to the active metabolite, oseltamivir carboxylate. It has an elimination half-life of approximately 8 h, primarily through the kidneys, and dose reduction is recommended for patients with an estimated creatinine clearance of less than 30 mL/min. Approximately 15 % of inhaled zanamivir reaches the bronchi and lungs. Excretion is primarily renal, but given its limited systemic bioavailability, there is no need to modify the dose in patients with renal insufficiency. The pulmonary half-life is 2.8 h. The most common toxicities reported with NI have been nausea and vomiting (approximately 15 % of patients). These side effects are usually mild and limited to the first days of treatment, although more serious side effects have been described. Oseltamivir has been linked to self-injury and delirium in pediatric populations, although no causal association could be demonstrated [42, 43]. The use of inhaled zanamivir has been associated with bronchospasm, sometimes severe or fatal, particularly in patients with underlying airways disease such as chronic obstructive pulmonary disease (COPD) or asthma [44].

Amantadine and rimantadine target the M2 protein of influenza A, which forms a proton channel in the viral membrane that is essential for viral replication. Amantadine is primarily excreted unchanged in the urine. In patients older than 65 years and in those with an estimated creatinine clearance of less than 50 mL/min, the daily dose should be reduced. Rimantadine is extensively metabolized in the liver, and dose reduction is recommended for patients with severe hepatic dysfunction, renal failure (creatinine clearance <10 mL/min), and the elderly. Central nervous system (CNS) side effects are well described with amantadine, particularly in elderly patients. In comparison, rimantadine is associated with a considerably reduced rate of CNS side effects [45].

## Clinical Outcomes

In mild to moderate uncomplicated disease, the reported benefits of early treatment (< 48 h of symptom onset) with NI have been a shorter duration and severity of flu-like symptoms, and a reduced duration of viral shedding [46–49]. More importantly, several trials and a few systematic reviews have shown that treatment with NI may reduce illness severity and the rate of lower respiratory tract complications [47, 50–52]. In a meta-analysis of 10 randomized-controlled trials, Kaiser et al. found that therapy with oseltamivir was effective in reducing the incidence of influenza-related lower respiratory tract complications that required antibiotic use (4.6 % for oseltamivir vs. 10.3 % for placebo, P < 0.001), independent of the presence of risk factors for complications [51]. More recently, Hernán et al. conducted another meta-analysis of 11 controlled trials, most of which were included in the previous meta-analysis by Kaiser et al. They found that treatment with oseltamivir significantly reduced influenza-related lower respiratory tract complications by 37 % (CI 95 % [18–52]) [52]. NI have also been reported to reduce the duration of hospitalization in severely-ill cases [53], and also to reduce influenza-related mortality [54]. During the 2009 H1N1 Influenza A pandemic, several observational studies of hospitalized and critically-ill patients reported that treatment with oseltamivir reduced disease severity, complications and mortality [55–57]. In a Chinese study of 1291 patients with confirmed H1N1 influenza A infection, oseltamivir reduced the risk of developing pneumonia, even when administered after the first 48 h of symptom onset (OR 0.12, 95 % CI [0.08–0.18]) [58]. In another retrospective study of 304 hematopoietic stem cell transplant recipients with influenza infection, 161 patients had H1N1 influenza A confirmed infection, and both early and delayed administration of antiviral therapy was shown to be beneficial in terms of decreased risk of lower respiratory tract compromise (OR = 0.04, 95 % CI [0–0.2] vs. OR = 0.14, 95 % CI [0–0.7]) [59]. An intravenous form of zanamivir is under development, and was made available during the 2009 H1N1 influenza A pandemic for severely ill patients with highly suspected or confirmed oseltamivir-resistant infection that could not tolerate inhaled zanamivir. Several studies reported favorable outcomes with the use of IV zanamivir [60–63].

During the 2009 swine influenza pandemic, the FDA briefly authorized the emergency use of peramivir, an investigational NI that is administered intravenously [64]. In one study, a single dose of 300 mg or 600 mg of IV peramivir significantly reduced the time to alleviation of symptoms compared with placebo (HR 0.68 and 0.67 for the 300 mg and 600 mg doses respectively) [65]. Laninamivir octanoate, another long acting NI in development, was non-inferior to a five-day course of oral oseltamivir in adults with seasonal influenza in one study [66]. Experience with avian influenza H5N1, which has caused sporadic cases since 2004 with an elevated mortality rate, is much more limited and recommendations for its treatment are in most cases extrapolated from trials of seasonal influenza. There are, however, reports that suggest reduced severity and mortality with the administration of oseltamivir in patients with avian influenza [67–69]. In patients with pneumonia or with clinical failure to standard regime with oseltamivir, a higher dose of 150 mg bid for 10 days should be considered [24, 67]. In regions with adamantine-susceptible strains, combination therapy with a NI and an adamantine may also be considered with pneumonia or clinical worsening [24]. Therapy with oseltamivir should be administered even in the late course of influenza A (H5N1) infection since viral replication is more prolonged than with seasonal influenza [24].

## Indications for Treatment

The United States Advisory Committee on Immunization Practices (ACIP) and the CDC have recently published updated guidelines for the treatment of patients with confirmed or suspected influenza virus infections caused by either pandemic H1N1 or seasonal strains [41••, 70••]. According to these recommendations, those individuals with severe disease (requiring hospitalization or with evidence of lower respiratory tract infection) or those at high risk for complications (Table 1) should receive antiviral therapy even after 48 h of symptom onset. Adults with mild illness without high risk conditions who are younger than 65 years of age do not require treatment. If such individuals present within the first 48 h of illness, antiviral treatment can be considered in order to reduce the duration of illness. In all cases, decisions to administer antiviral medications for influenza treatment should be based on clinical and epidemiologic grounds, and never be delayed because of confirmatory laboratory tests [41••]. The usual dosing of oseltamivir for the treatment of influenza is 75 mg orally twice daily and of zanamivir is 10 mg (2 inhalations) twice daily. The recommended duration for antiviral treatment is 5 days, although longer treatment courses for patients who fail to improve after 5 days of treatment can be considered [70••]. Also, doubling the dose of oseltamivir to 150 mg orally twice daily has been suggested to be beneficial for some severely ill patients with H5N1 avian influenza [24, 67]. The FDA licensed oseltamivir for use in children 1 year of age and older in a liquid formulation at a dosage of 2 mg/kg/dose twice daily for 5 days. Zanamivir is not approved for use in children under 5. Oseltamivir and zanamivir are Pregnancy Category C drugs, but since influenza causes more severe disease and an increased rate of mortality among pregnant women, they should receive antiviral therapy with oseltamivir when indicated, since the potential benefit outweighs the theoretical risk to the fetus (Table 1).

## Resistance

Although oseltamivir-resistant seasonal H1N1 influenza A viruses have been identified since 2007 (the H274Y mutation) [71], in 2009 the CDC reported that most circulating strains of the novel H1N1 influenza A virus were sensitive to the NI, oseltamivir and zanamivir, but that nearly all strains were resistant to the amantadines [41••, 72]. Consequently, the ACIP has advised against the use of M2 inhibitors for treatment of influenza, except in selected circumstances [41••].

## Additional Management Strategies

It is recommended that patients with pandemic H1N1 influenza A who develop pneumonia be treated empirically for CAP according to published evidence-based guidelines, given the risk of secondary bacterial pneumonia [73]. In the presence of profound hypoxemia that has been refractory to routine mechanical ventilation, salvage therapies include neuromuscular blockade, inhaled nitric oxide, high-frequency oscillatory ventilation, extracorporeal membrane oxygenation (ECMO), and prone positioning ventilation [74•, 75]. Corticosteroids should not be used routinely, but may be considered for septic shock with suspected adrenal insufficiency requiring vasopressors [76]. Therapy for influenza-associated ARDS should be based upon published evidence-based guidelines for sepsis-associated ARDS, specifically including lung protective mechanical ventilation strategies [76].

## Prevention

The CDC recommends routine annual influenza vaccination for all persons 6 months of age and older. When vaccine supply is limited, vaccination efforts should focus on those groups with health conditions associated with increased risk of influenza complications [40••]. Antiviral drugs should not be used as a substitute for influenza vaccination. Their adjunctive use is appropriate in certain targeted populations at high risk for complications of influenza who are close contacts with suspected or confirmed cases [Table 1]. Post-exposure prophylaxis should only be used when antivirals can be started within 48 h of the most recent exposure. Recommended duration is 7 days after exposure, and the CDC recommends a minimum of 2 weeks for control of influenza outbreaks in long–term care facilities (i.e.*,* nursing homes with elderly) and hospitals [70••]. The choice to offer post-exposure prophylaxis to otherwise healthy unvaccinated adults should be weighed against the risk of promoting antiviral drug resistance [41••, 1••].

## Conclusions

A potentially fatal complication of influenza infection is the involvement of the lower respiratory tract caused directly by the influenza virus, and the development of secondary bacterial pneumonia. In these cases, and in patients who are at an increased risk for influenza infection complications, confirmation of etiology by laboratory testing is required in order to guide the initiation and duration of antiviral treatment, and for the implementation of infection control measures and surveillance. Recently, the emergence of novel influenza A strains that carry the risk for more severe disease regardless of age or previous health status, has prompted the development of quick and reliable laboratory tests in an effort to optimize their management and reduce morbidity and mortality. Pneumonia related to the 2009 influenza A pandemic was found in many cases to be rapidly progressive, leading to respiratory failure which in many cases was underestimated by commonly used pneumonia severity scores. Given the limited sensitivity of RIDT and immunofluorescence assays, confirmation of pandemic H1N1 influenza A infection can only be made by rRT-PCR or viral culture. Although annual immunization is the most important preventive measure, NI are the agents of choice for chemoprophylaxis in selected high risk patients, and for treatment. Treatment with NI beyond 48 h of symptoms should be considered only for patients with severe disease.
